# Transcriptional Programs Driving Shear Stress-Induced Differentiation of Kidney Proximal Tubule Cells in Culture

**DOI:** 10.3389/fphys.2020.587358

**Published:** 2020-10-30

**Authors:** Hyun Jung Park, Zhenjiang Fan, Yulong Bai, Qidong Ren, Youssef Rbaibi, Kimberly R. Long, Megan L. Gliozzi, Natalie Rittenhouse, Joseph D. Locker, Amanda C. Poholek, Ora A. Weisz

**Affiliations:** ^1^Department of Human Genetics, Graduate School of Public Health, University of Pittsburgh, Pittsburgh, PA, United States; ^2^Department of Computer Science, University of Pittsburgh, Pittsburgh, PA, United States; ^3^School of Medicine, Tsinghua University, Beijing, China; ^4^Renal-Electrolyte Division, University of Pittsburgh School of Medicine, Pittsburgh, PA, United States; ^5^Division of Pediatric Rheumatology, Department of Pediatrics, University of Pittsburgh School of Medicine, Pittsburgh, PA, United States; ^6^Department of Pathology, University of Pittsburgh School of Medicine, Pittsburgh, PA, United States

**Keywords:** kidney, proximal tubule, endocytosis, cell culture, shear stress

## Abstract

Cultured cell models are an essential complement to dissecting kidney proximal tubule (PT) function in health and disease but do not fully recapitulate key features of this nephron segment. We recently determined that culture of opossum kidney (OK) cells under continuous orbital shear stress (OSS) significantly augments their morphological and functional resemblance to PTs *in vivo*. Here we used RNASeq to identify temporal transcriptional changes upon cell culture under static or shear stress conditions. Comparison of gene expression in cells cultured under static or OSS conditions with a database of rat nephron segment gene expression confirms that OK cells cultured under OSS are more similar to the PT *in vivo* compared with cells maintained under static conditions. Both improved oxygenation and mechanosensitive stimuli contribute to the enhanced differentiation in these cells, and we identified temporal changes in gene expression of known mechanosensitive targets. We observed changes in mRNA and protein levels of membrane trafficking components that may contribute to the enhanced endocytic capacity of cells cultured under OSS. Our data reveal pathways that may be critical for PT differentiation *in vivo* and validate the utility of this improved cell culture model as a tool to study PT function.

## Introduction

Cells lining the kidney proximal tubule (PT) consistently recover ∼70% of water, sodium, chloride, and other solutes entering the tubule lumen as well as essentially all of the glucose and filtered proteins (Alan et al., 2015). To enable the extraordinary and rapidly adaptive transport and endocytic demands required for proper function in the face of frequent changes in glomerular filtration rate, PT cells maintain highly structured apical and basolateral plasma membrane domains, a capacious apical endocytic pathway, and highly efficient metabolic processing of lactose and fatty acid fuels (Christensen et al., 2012; Eshbach and Weisz, 2017). Dissecting the intricacies of PT function *in vivo* is challenged by the inaccessibility of this nephron segment as well as the multitude of neighboring cell types that influence renal responses to changes in flow. Conversely, studying PT function in cell culture has been hampered by the paucity of well-differentiated immortalized cell lines or primary cell culture models.

In terms of their similarity to PTs *in vivo*, opossum kidney (OK) cells currently represent the best *in vitro* model in which to study transport and endocytic functions of this nephron segment. When cultured on permeable supports, these cells differentiate into a polarized monolayer that retains expression of ion transporters and endocytic receptors essential for PT function (Malström et al., 1987; Gekle et al., 1996; Murer et al., 2000; Zhai et al., 2000). Physiologic regulatory mechanisms are also largely preserved in this cell line. For example, unlike other PT cell models, OK cells uniquely retain the regulatory cascade that directs sodium dependent phosphate transport in response to parathyroid hormone (Malmström and Murer, 1986). Moreover, OK cells internalize albumin and other filtered molecules with high efficiency compared to other PT cell lines and have been a useful model in which to study PT endocytosis (Gekle et al., 1995, 1996, 1997, 1998; Raghavan et al., 2014; Long et al., 2020; Ren et al., 2020).

Despite their utility, OK cells cultured under standard conditions do not fully recapitulate key aspects of PT structure and function. However, we recently determined that OK cells plated on permeable supports and exposed for 96 h to orbital shear stress (OSS, 146 rpm on a rotating shaker) were taller, proliferated more rapidly, and elaborated more microvilli compared with cells cultured under static conditions (Long et al., 2017). Moreover, numerous subapical vesicular and tubular structures were evident in cells cultured under OSS, consistent with a dramatic expansion of the apical endocytic pathway. Additionally, as *in vivo*, these cells developed elaborately folded basolateral membranes that surround mitochondria to provide enhanced surface area and energy for Na^+^/K^+^-ATPase-mediated sodium transport. Western blotting of cell lysates revealed two- to four-fold increases in the expression of Na^+^/K^+^-ATPase (α1 subunit), megalin, and Rab11a in cells exposed to OSS. Consistent with their high metabolic demands, cells exposed to OSS exhibited dramatically increased numbers of lysosomes and mitochondria compared with cells cultured under static conditions, and maintained significantly higher levels of adenine nucleotides, including ATP and NADH (Long et al., 2017).

We previously assembled the transcriptome of OK cells cultured on Transwell supports under static conditions (Eshbach et al., 2017). To identify transcriptional changes that lead to the remarkable differentiation we observed in cells exposed to OSS, we performed a time course RNASeq study using RNA isolated from OK cells exposed to OSS or maintained under static conditions for 0-96 h. Previous analysis of a subset of these data, together with biochemical and metabolomic approaches, revealed dramatic metabolic changes upon cell exposure to OSS, consistent with a shift from glycolytic to gluconeogenic metabolism characteristic of the PT *in vivo* (Ren et al., 2019). These changes likely reflect the increased cellular access to O_2_ when cells are cultured under OSS. Additionally, by comparing the spatial profile of Na^+^/K^+^-ATPase distribution and endocytic capacity across the radius of the permeable supports, we concluded that mechanosensitive stimuli also contribute to the enhanced PT functions of cells cultured under OSS (Ren et al., 2019). Here we performed a more comprehensive analysis of our RNASeq database to (1) identify the temporal sequence of pathways that induce OSS-mediated differentiation of OK cells; (2) identify ion transport and membrane trafficking proteins whose transcriptional expression correlates with enhanced PT morphology and function; and (3) compare the transcriptional profile of our optimally differentiated cell culture with that of microdissected rat PT nephron segments. Our data support the use of this cell culture model as a useful system in which to study the regulation of PT transport and membrane trafficking pathways.

## Materials and Methods

### Cell Culture and RNA Seq

Cell culture and RNA Seq were described in Ren et al. (2019). Briefly, 4 × 10^5^ wild type OK cells (RRID:CVCL_0472) were plated at superconfluence on 12 mm permeable supports in 0.5 mL apical and 1.5 mL basolateral DMEM/F12 medium (Sigma D6421) with 10% FBS (Atlanta Biologicals) and 5 mM GlutaMAX (Gibco). After 18 h (*t* = 0 h), a subset of the filters were transferred to an orbital platform shaker rotating at 146 rpm (OSS) and the incubation continued. Cells were collected using Accutase (BD Biosciences) and RNA was extracted using the Ambion PureLink RNA mini kit (Thermo Fisher Scientific) at 12, 48, and 96 h after transferring cells to OSS and at 0, 48, and 96 h from cells maintained under static conditions. RNA from three experiments was combined to create each sample, and three independent samples were sequenced for each time point. Library preparation was performed using the TruSEQ Stranded Total RNA Sample Preparation Kit (Illumina) according to manufacturer’s instructions. Following removal of ribosomal RNA, the remaining RNA was fragmented for 8 min, followed by reverse transcription. Double stranded cDNA was subjected to 3′ adenylation and ligation of sequencing adapters. Sequencing was carried out on a NextSeq 500 (Illumina) to generate 75 bp paired-end reads. An average of 80 million paired reads were analyzed per sample. Raw sequence reads were trimmed of adapter sequences using cutadapt (Martin, 2011) and mapped to the *Monodelphis domestica* reference genome (MonDom5) using TopHat2 (Kim et al., 2013), allowing for a base-pair mismatch value of 6. Prior to calculating raw count values with the Subread package feature Counts (Liao et al., 2014), reads aligned to mitochondrial genes and ribosomal RNA were removed. Heat maps and comparison of membrane trafficking protein expression was performed after normalizing the dataset to account for differences in total reads. Sequencing files have been deposited in GEO (GSE155315) and normalized gene quantification for all samples is provided in Supplementary Table 1.

### Albumin Uptake and Fluorescence Staining

Opossum kidney cells cultured on permeable supports for 0h or for 96h under OSS or static conditions were incubated with 40 μg/mL apically added AlexaFluor-647 albumin (Invitrogen A34785) for 15 min, then washed twice in warm PBS/+Ca^2+^/+Mg^2+^ (Sigma D8662) and fixed in 4% paraformaldehyde in 100 mM sodium cacodylate for 15 min at ambient temperature. After two washes in PBS, filters were quenched (PBS, 20 mM glycine, and 75 mM ammonium chloride) for 5 min. Filters were blocked with PBS, 1% BSA, and 0.1% saponin for 30 min and incubated for 30 min with Rhodamine Phalloidin (Cytoskeleton, PHDR1) diluted in wash buffer [PBS, 0.5% BSA, and 0.025% (v/v) saponin]. Filters were washed three times in wash buffer and mounted onto glass slides with ProLong Gold antifade reagent (Molecular Probes, P36935). Filters were imaged on a Leica TCA SP5 confocal microscope using the PL APO CS × 63 objective. Images were collected and processed using identical conditions. Maximum projections of representative fields are shown.

### Condition-Stratified Differential Expression Analysis

In 18 samples in our data, we retained the genes with CPM (counts per million) greater than 1 in at least 6 samples. Then, we stratified the samples by biological condition, OSS or static. In each stratum, we normalized the library size using scaling factors calculated by a trimmed mean of M-values (TMM) between each pair of samples, which is implemented by edgeR. Then, with the normalized data, we fit a generalized linear model to identify differentially expressed genes. The only predictor in our model is time point, and the dispersion parameter is estimated using quantile-adjusted conditional maximum likelihood (qCML) method, which is also implemented by edgeR. The contrasts are made along the time series (start vs. intermediate; intermediate vs. end), quasi-likelihood *F*-tests are performed to estimate the significance. Additionally, we conducted differential expression analyses between OSS and static at the start and the end time points separately. All the statistical methods used are same to previous DE analysis, except the only predictor in the general linear model (GLM) is biological condition, not time point. Based on this model, we selected differentially expressed genes with false discovery rate (FDR) < 0.05.

### Pathway Analysis

Pathway analysis was mainly performed using Ingenuity Pathway Analysis (IPA). Depending on our interest, we used Disease and Biological Functions or Canonical Pathway terms. Ingenuity Pathway Analysis comparison analysis that compares the results of two IPA differential analyses was used to elucidate consecutive molecular changes across three time points (e.g., 96 h vs. 48 h vs. 12 h in Figure 2) or compare two later time points (e.g., 96 h OSS and 96 h Static in Figure 3) against the same baseline (0 h Static). Where appropriate, the IPA activation z-score, which represents the bias in gene regulation, was used to predict whether a given pathway is activated or inactivated relative to the compared condition. Otherwise, we used a canonical measure representing statistical significance, *P*-value. IPA calculates *P*-value from a Right-Tailed Fisher’s Exact Test, which reflects the likelihood that the overlap between our observation and each IPA knowledgebase pathway is due to random chance. *P*-values were further corrected for multiple testing with Benjamini–Hochberg.

### T-Distributed Stochastic Neighbor Embedding Analysis

We used a curated set of highly expressed genes in human and rat nephron segments described in Eshbach et al. (2017) to compare the expression of our data (96 h OSS and 96 h Static) with that of S1, S2, and S3 PT segments reported in Lee et al. (2015) We extracted two dimensions of the embedded space from t-distributed Stochastic Neighbor Embedding (tSNE) on which to place our data, S1, S2 and S3 PT.

### Genes for the Annotated Gene Sets (e.g., Signaling Pathways) From MSigDB

To retrieve genes involved in biochemical processes of interest (e.g., mechanosensation), we used regulatory target gene sets (C3) defined in MSigDB version 7.1 (Subramanian et al., 2005). We used the UCSC table browser to retrieve the gene identification (ID), symbol, and description associated with GRCh38/hg38 and mm10 assembly, GENCODE v32 track. The columns in the matching table are from kgXref table which links known gene ID and their reference. The kgID column contains the ensemble transcript ID. The Gene Symbol column contains official gene symbol and the Description column contains the full gene names and the source databases.

### Heatmap Log Transformation and Row-Wise Normalization

Gene expression values were first log-transformed and the values within each row were normalized to sum to 1 to generate heat maps. The top 10% of the genes in terms of the standard deviation of expression across the conditions and with an average count of >100 are plotted.

### Western Blotting

Equivalent amounts of cell lysate (10 μg) extracted from cells cultured under static or OSS conditions for 96h were western blotted with the following antibodies: Dab2 (D709T, Cell Signaling Technology #12906); Caveolin-1 (Cell Signaling Technology #3238); Rab11a (Abcam #ab65200); and Dynamin-2 (Hudy-1, EMD Millipore #MABT188). Band intensities were quantified using and background subtracted. Intensities of OSS-exposed samples were normalized to control (Static) values (set at 100%) and significance was assessed separately for each antibody in GraphPad Prism software using a one sample *t*-test.

## Results

### OSS-Induced Changes in PT Cell Gene Expression

**FIGURE 1:**
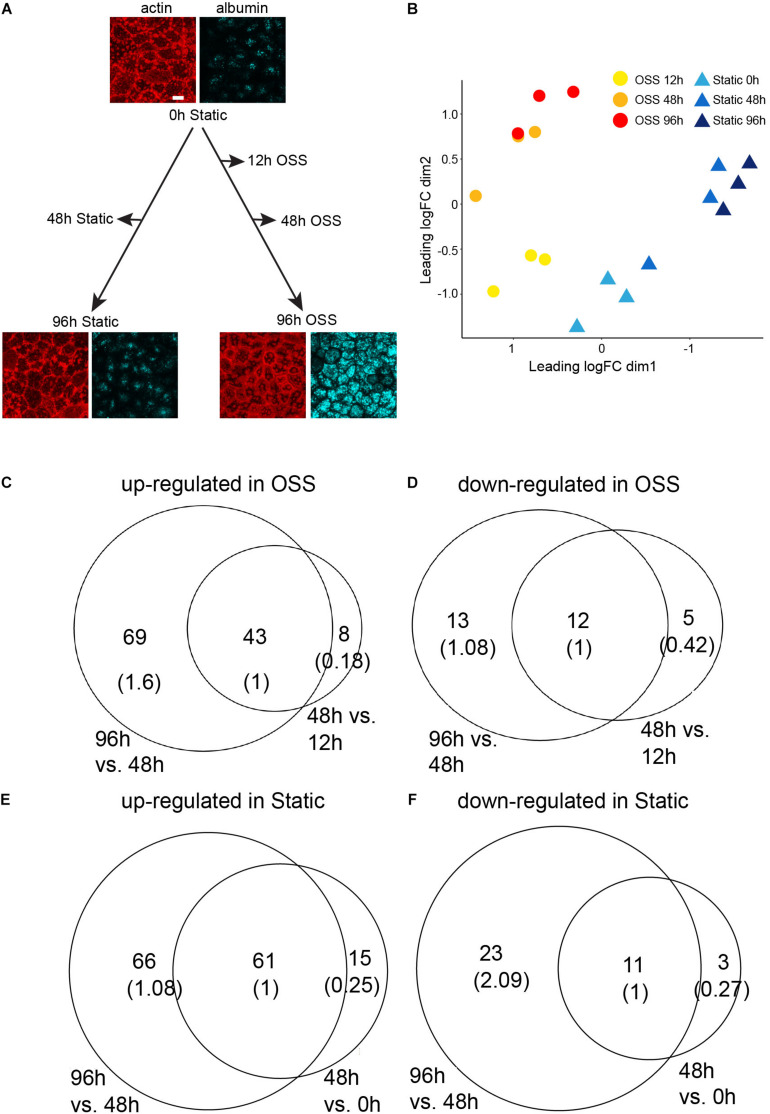
RNASeq experimental design and analysis on differentially expressed genes. **(A)** OK cells plated on permeable filter supports and the following day (*t* = 0 h) transferred to orbital shear stress (OSS) or maintained under static conditions. RNA was harvested at the indicated time points. The images show actin staining (red) and albumin uptake (blue) of cells at 0 h and after 96 h culture under static or OSS conditions. Scale bar: 10 μm. **(B)** MDS visualization of the three biological replicates from each condition based on their gene expression profiles. The profiles are further grouped by the time point at which the RNA-Seq data were harvested (0, 48, 96 h for static and 12, 48, 96 h for OSS). The number of up- **(C)** and down- **(D)** regulated genes in cells exposed to OSS for 48 h vs. 12 h (right circle) and in 96 h vs. 48 h (left circle) with the overlap normalized to 1 in parentheses. The number of up- **(E)** and down- **(F)** regulated genes in cells cultured under static conditions for 48 h vs. 0 h (right circle) and for 96 h vs. 48 h (left circle).

To identify transcriptional changes that lead to the remarkable differentiation we observed in cells exposed to OSS, OK cells were plated on permeable supports, and the following day (*t* = 0h) shifted to OSS for 12, 48, and 96 h or maintained under static conditions over this period (0, 48, and 96 h) before harvesting RNA. Similarly plated samples cultured for 0 h or for 96 h under OSS or static conditions were incubated with apically added AlexaFluor-647 albumin for 15 min, then fixed and stained with rhodamine-phalloidin to confirm the increase in apical endocytic activity and buttressed cytoskeletal structure previously observed under OSS (Figure 1A; Long et al., 2017). RNA from three independent time courses was pooled for each sample, and three samples for each condition were sequenced. Multidimensional scaling (MDS) based on the gene expression profile between datasets for each condition (Figure 1B) supports validity of the datasets. First, based on the gene expression profiles, the three biological replicates for each condition are closer to each other than to those of the other conditions. Second, the gene expression profiles change progressively over time.

Cells exposed to OSS for 12 h already had a significantly different transcription profile compared with the starting cell population (0 h). We identified 41 transcripts significantly (FDR < 0.05) down-regulated upon exposure to OSS for 12 h compared with cells recovered at 0h. Culture for 48 or 96 h under OSS resulted in further divergence in transcripts compared with the starting (0 h) cell population. There were 51 transcripts upregulated and 17 downregulated by 48 h of culture and an additional 69 upregulated and 13 downregulated by 96 h OSS (Figures 1C,D, respectively). For cells maintained under static conditions, we identified 76 upregulated and 14 downregulated transcripts at 48 h compared to 0 h and an additional 66 upregulated and 23 downregulated transcripts at 96 h (Figures 1E,F, respectively).

Transcripts that were rapidly downregulated within 12 h of OSS are shown in Table 1. The most highly downregulated transcript was for LRP2BP, a megalin-binding protein recently demonstrated to promote cell migration in vascular smooth muscle cells (Zhang et al., 2018). Significantly downregulated transcripts also encoded proteins commonly associated with kidney function and disease, including adrenomedullin (*ADM*), klotho (*KL*), fibrocystin-L (*PKHD1L1*), and angiopoietin 1 (*ANGPT1*). Ingenuity Pathway Analysis analysis identified 76 pathways consistent with changes in cell differentiation, transport, protein trafficking, and metabolism that were significantly altered within 12h of exposure to OSS (Supplementary Table 2). A subset of these are shown in Table 2.

**TABLE 1:** Transcripts downregulated after 12 h exposure to OSS.

Gene	logFC	logCPM	F statistic	*P*-Value	FDR	Protein
LRP2BP	–3.5565	4.845489	335.3451	5.22E-08	0.000463	Lrp2 binding protein
CA9	–4.5667	3.931135	188.9926	5.27E-07	0.002055	Carbonic anhydrase IX
DHRS9	–4.5225	3.627682	124.1449	2.79E-06	0.003282	Dehydrogenase/reductase (SDR family) member 9
BLK	–4.7370	3.865411	123.433	2.85E-06	0.003282	B lymphoid kinase
SUSD4	–3.4992	3.567026	111.0262	4.31E-06	0.003282	Sushi domain containing 4
GPR37	–3.2780	2.435743	108.3623	4.74E-06	0.003282	G protein-coupled receptor 37
ERO1A	–2.0756	7.235736	95.66971	7.67E-06	0.003564	Endoplasmic reticulum oxidoreductase 1 alpha
CALML3	–2.8456	6.088465	89.46132	9.93E-06	0.003974	Calmodulin-like 3
SCARA3	–2.2841	3.424677	80.82479	1.46E-05	0.003983	Scavenger receptor class A, member 3
ADM	–3.6496	5.58256	72.16275	2.25E-05	0.004513	Adrenomedullin
KCNAB2	–2.7174	4.500963	65.95435	3.15E-05	0.005386	Potassium voltage-gated channel, shaker-related subfamily, beta member 2
KL	–3.5811	–0.49906	65.22774	3.28E-05	0.005386	Klotho
PKHD1L1	–2.7306	3.91887	63.25991	3.68E-05	0.005509	Fibrocystin-L; polycystic kidney and hepatic disease 1-like 1
ANGPT1	–2.0976	3.425352	56.54254	5.57E-05	0.006502	Angiopoietin 1
CPNE5	–3.7010	0.135471	56.12173	5.72E-05	0.006502	Copine V
ST3GAL1	–2.7728	5.076463	54.56327	6.35E-05	0.006773	ST3 beta-galactoside alpha-2,3-sialyltransferase 1
IGFBP3	–3.4093	5.386319	53.92867	6.62E-05	0.006985	Insulin-like growth factor binding protein 3
NDRG2	–2.1087	5.667499	52.54419	7.28E-05	0.007118	N-myc downstream regulated gene 2
ADAMTS13	–3.2598	1.860187	50.62773	8.33E-05	0.007118	A disintegrin-like and metallopeptidase
SPATA17	–2.0973	1.079873	48.33872	9.85E-05	0.007626	Spermatogenesis associated 17
ENO2	–2.3957	2.921824	47.56873	0.000104	0.007705	Enolase 2
SPINT1	–2.2497	3.483428	47.07817	0.000108	0.007802	Serine protease inhibitor, Kunitz type 1
TNFRSF19	–3.9773	0.061145	46.90267	0.00011	0.007823	Tumor necrosis factor receptor superfamily, member 19
REPS2	–2.1065	4.149959	43.27926	0.000146	0.008666	RALBP1 associated Eps domain containing protein 2
GRHL3	–2.5177	1.412018	42.64034	0.000154	0.008744	Grainyhead like transcription factor 3
KCNK13	–2.6555	–0.46147	42.50643	0.000156	0.008744	Potassium channel, subfamily K, member 13
TRIM63	–2.0653	3.552744	38.78596	0.000215	0.010181	Tripartite motif-containing 63
KANK3	–2.2144	3.613935	38.62101	0.000218	0.010181	KN motif and ankyrin repeat domains 3
PCSK1	–3.2134	1.50546	38.48542	0.000221	0.010191	Proprotein convertase subtilisin/kexin type 1
UBA7	–2.1927	1.502012	33.93303	0.000341	0.011741	Ubiquitin-like modifier activating enzyme 7
LTBP1	–2.0196	2.798	33.23028	0.000367	0.012115	Latent transforming growth factor beta binding protein 1
SRMS	–2.4056	0.549291	30.55573	0.000486	0.013434	Src-related kinase lacking C-terminal regulatory tyrosine and N-terminal myristylation sites
ADGRF5	–3.4251	–1.03889	29.24985	0.000563	0.014112	Adhesion G protein-coupled receptor F5
ANKRD1	–2.2238	0.282321	27.70426	0.000673	0.015169	Ankyrin repeat domain 1
P2RY6	–2.312	–0.75905	18.98575	0.002214	0.025303	Pyrimidinergic receptor P2Y6
ANKRD37	–2.97	0.438142	18.57396	0.002364	0.026339	Ankyrin repeat domain 37
TRIM55	–2.0308	–0.26692	16.5463	0.003321	0.030642	Tripartite motif-containing 55
TMEM52B	–2.0286	1.403966	16.48555	0.003356	0.03081	Transmembrane protein 52B
CYP4F22	–3.3780	–1.37273	15.92283	0.003708	0.032597	Cytochrome P450 family 4 subfamily F member 22
EFHC2	–2.0731	–0.22658	14.11734	0.005198	0.03926	EF-hand domain (C-terminal) containing 2
ENPP2	–2.0774	4.911872	12.37982	0.007405	0.048088	Ectonucleotide pyrophosphatase/phosphodiesterase 2

*The log2-transformed fold-change (logFC), log2-transformed counts per million (logCPM), *F*-statistic, *P*-value, and false discovery rate (FDR) for differential expression of genes estimated by edgeR is shown, along with the name of the protein encoded by each gene.*

**TABLE 2:** IPA Pathway Analysis: 0 h vs. 12 h OSS (selected pathways).

Category	P-value (range)
Organ development	3E-06-1.98E-02
Tissue morphology	1.73E-04-1.97E-02
Cellular movement	1.82E-04-2E-02
Molecular transport	2.07E-04-1.79E-02
Protein trafficking	2.07E-04-1.61E-02
Amino acid metabolism	4.47E-04-1.08E-02
Post-Translational modification	4.47E-04-1.61E-02
Inflammatory response	1.37E-03-1.97E-02
Carbohydrate metabolism	1.8E-03-1.79E-02
Cell cycle	1.8E-03-1.26E-02
Lipid metabolism	1.8E-03-1.79E-02
Nucleic acid metabolism	1.8E-03-8.6E-03
Renal and urological system development and function	1.8E-03-1.98E-02
Protein folding	1.43E-02-1.61E-02
Vitamin and mineral metabolism	1.79E-02-1.79E-02

*A subset of the 76 pathways identified by IPA comparison of OK cells cultured under orbital shear stress (OSS) for 0 vs. 12 h is shown, along with the range in *P*-values. Supplementary Table S2 shows all pathways and genes therein.*

### Temporal Changes in OSS-Induced Transcription

**FIGURE 2:**
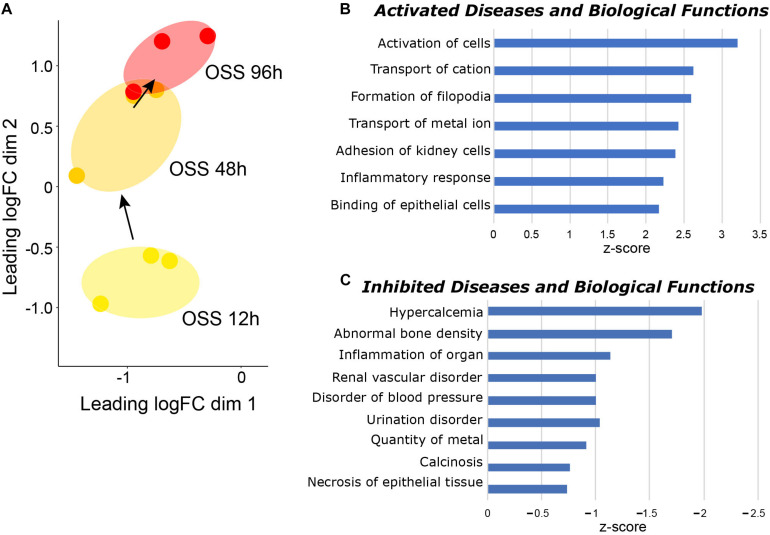
Temporal gene expression changes in cells exposed to OSS. **(A)** MDS visualization of the gene expression profiles using IPA comparison analysis (96 h OSS vs. 48 h OSS and 48 h OSS vs. 12 h OSS). IPA *Diseases and Biological Function* terms that are selectively **(B)** enriched and **(C)** de-enriched at later times (96 h OSS vs. 48 h OSS) compared with early times of OK cell culture under OSS (48 h OSS vs. 12 h OSS) are plotted in order of increasing activation z-score.

Paired datasets were compared using IPA to identify pathways that were temporally engaged or de-enriched upon prolonged exposure to OSS compared with shorter periods (Supplementary Table 3). To this end, we identified significant changes in IPA *Diseases and Biological Functions* pathways between cells exposed to OSS for 48 h vs. 12 h and for 96 h vs. 48 h (Figure 2A). Numerous pathways relating to ion transport and cell adhesion were selectively activated between 48 and 96 h but not between 12 and 48 h OSS (Figure 2B), suggesting that these represent later stages of differentiation. Moreover, pathways that were selectively de-enriched upon prolonged exposure to OSS were largely associated with kidney disease (e.g., urination disorder, calcinosis, disorder of blood pressure, and inflammation (Figure 2C). We interpret this to mean that between 48 and 96 h of exposure to OSS, OK cells differentiation increasingly recapitulates normal PT physiology compared with exposure for <48 h.

### Transcriptional Divergence of Cells Cultured Under OSS vs. Static Conditions

Our prior studies demonstrate that the function, metabolism, and morphology of OK cells cultured under OSS more closely resembles that of PT cells *in vivo* (Long et al., 2017; Ren et al., 2019). To identify gene expression changes that contribute to this enhanced differentiation and assess how these compare with known gene expression profiles of PTs *in vivo*, we first compared the transcriptional profile of cells cultured for 96h under static vs. OSS conditions. A total of 339 genes were differentially expressed in cells cultured under OSS vs. static conditions (104 upregulated, 235 downregulated). A list of these genes and the proteins they encode is shown in Supplementary Table 4.

**FIGURE 3:**
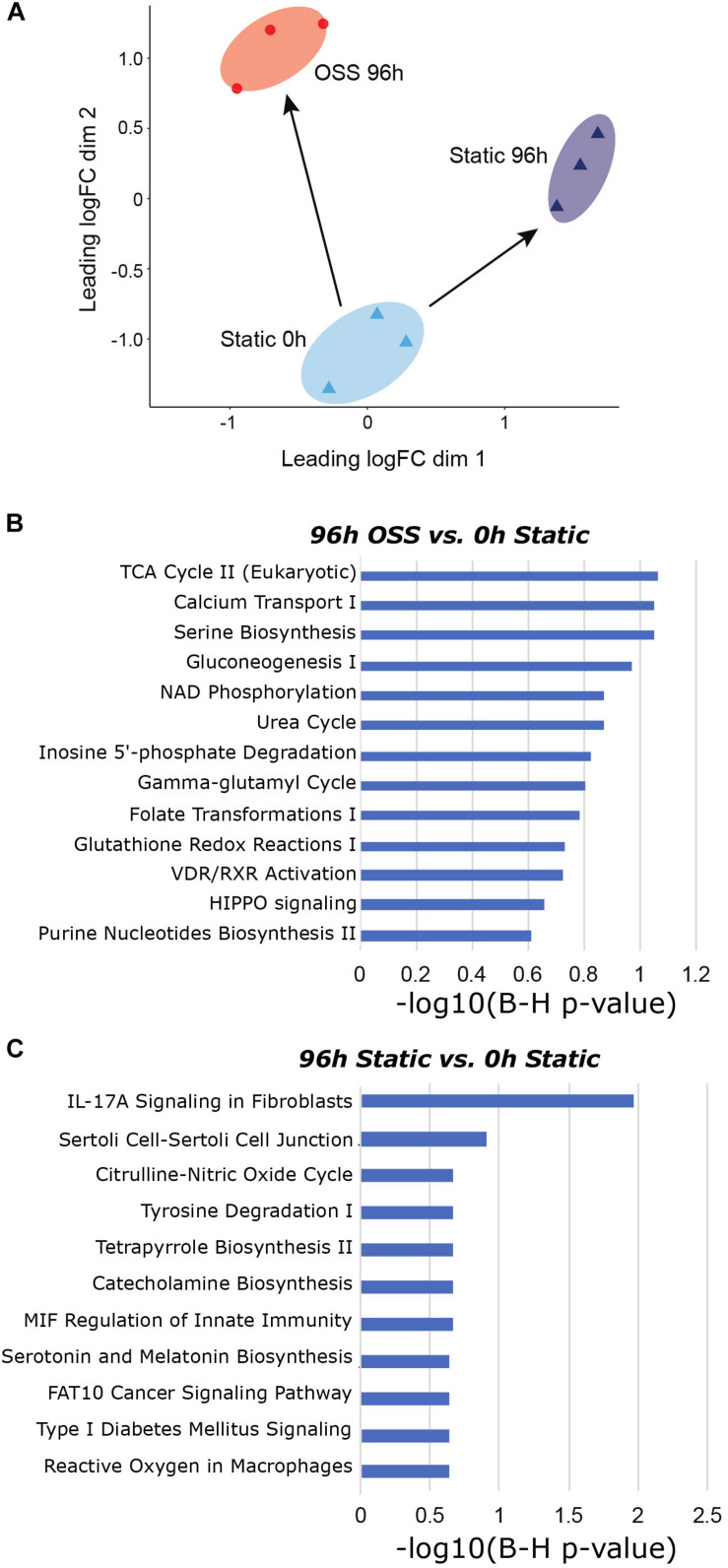
Gene expression changes between OSS and static at 96h in reference to 0h static. **(A)** MDS visualization of the gene expression profiles analyzed using IPA comparison analysis (96 h OSS vs. 0 h Static and 96 h Static vs. 0 h Static). **(B)** IPA analysis results using *Canonical Pathway* terms enriched in 96 h OSS vs. 0 h Static. **(C)** IPA analysis results against *Canonical Pathway* terms enriched in 96 h Static vs. 0 h Static. [*P*-value = −log 10(B-H *P*-value)].

Additionally, we employed IPA comparison analysis to identify those pathways differentially altered in cells cultured for 96 h under OSS or under static conditions compared with the starting population at 0 h (Figure 3A). A selected list of canonical pathways that were differentially upregulated in cells cultured under OSS (Figure 3B) or static conditions (Figure 3C) for 96 h vs. 0 h is provided. The full list of *Canonical* and *Diseases and Biological Functions* pathways identified in this analysis is provided in Supplementary Table 5. Nearly all pathways that were exclusively activated by culture under OSS were metabolicaly related. Of interest, the HIPPO signaling pathway, which controls cell proliferation vs. apoptosis was also identified as a selectively enriched pathway in cells cultured under OSS. Eleven pathways were selectively enriched upon cell culture under static conditions compared with OSS, and many of these were associated with non-renal cells (e.g., catecholamine biosynthesis, serotonin and melatonin biosynthesis, and pathways related to immune responses, nitric oxide production, and cancer.

### Culture Under OSS Enhances the PT-Specific Transcriptional Profile of OK Cells

**FIGURE 4:**
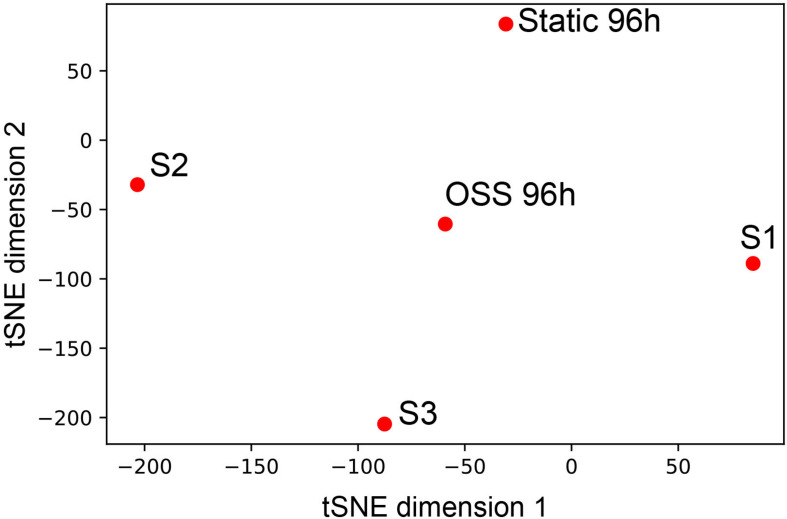
Similarity of 96 h OSS and 96 h Static to gene expression in microdissected rat nephron segments. tSNE visualization of 96 h OSS and 96 h Static and S1, S2, and S3 rat PT cells from Lee et al. (2015) based on expression information of the genes highly expressed in human and rat nephron segments (see section “Materials and Methods”).

Based on our functional and morphological studies, we predicted that the transcriptional profile of cells cultured for 96h under OSS would more closely resemble that of PT segments *in vivo*. To this end, we compared our data with deep sequencing data obtained from microdissected rat nephron segments for the genes that were highly expressed in any of the segments, including S1, S2, and S3 (Lee et al., 2015). T-distributed Stochastic Neighbor Embedding analysis of the data reveals that the 96 h OSS gene expression profile is consistently closer to S1, S2, and S3 than the 96 h static profile (Figure 4). The 96 h OSS data was roughly equidistant from all three PT segments, suggesting that these cells do not especially resemble S1, S2, or S3 segments but represent a more generic model of PT function.

### Oxygen Availability and Mechanosensation Differentially Drive OSS-Induced OK Cell Differentiation

**FIGURE 5:**
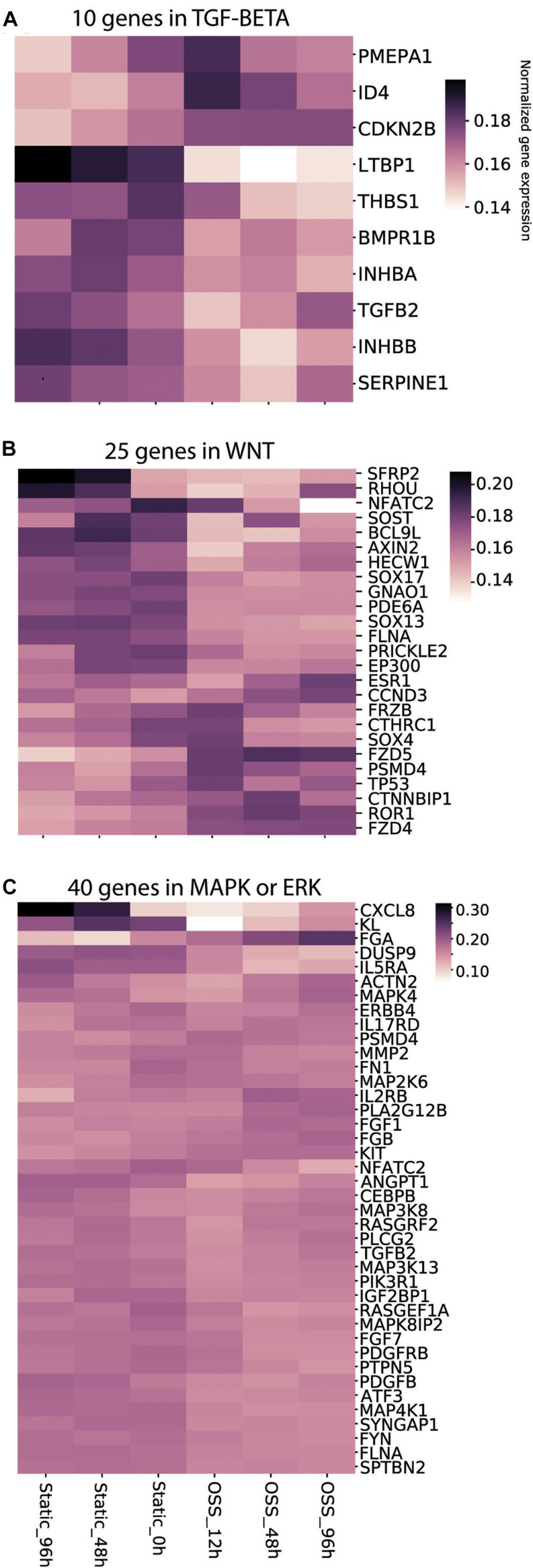
Culture-dependent temporal gene expression changes in mechanosensitive signaling pathways. Heat maps of the gene expression profile of gene sets defined using keywords **(A)** TGF-β, **(B)** Wnt, and **(C)** MAPK/ERK are plotted for cells incubated for 96, 48, and 0 h under Static conditions or 12, 48, and 96 h under OSS.

We previously showed that both metabolic changes resulting from increased exposure to O_2_ and mechanosensitive cues contribute to the enhanced differentiation of cells treated for 96 h under OSS (Ren et al., 2019). Whereas changes in metabolic pathways were readily identified, mechanosensitive changes in PT transcription have not been extensively studied. Transcriptional changes upon exposing immortalized mouse PT epithelial cells (PTECs) for 6h to OSS using a cone-plate device were recently reported (Kunnen et al., 2018). That study identified the TGF-β, MAPK/ERK, and Wnt signaling pathways as core pathways upregulated upon exposure to OSS, with changes in the transcription of genes involved in cell–matrix interactions, cytoskeletal dynamics, glycolysis, and cholesterol metabolism. Because the cone-plate apparatus used to generate shear stress in that study limits O_2_ access to the plated cells, we reasoned that the identified genes and pathways would be skewed to reflect targets of mechanical stimulation (as opposed to changes in oxygenation). We therefore used their list of genes/pathways as templates to gauge the profile of mechanosensitive transcription in our cells (see section “Materials and Methods”). Figure 5 shows the temporal changes in the top 10% of genes within the TGF-β (Figure 5A), Wnt (Figure 5B), and MAPK/ERK (Figure 5C) pathways. Changes in genes related to cellular matrix, cholesterol metabolism and the cytoskeleton are provided in Supplementary Figure 1. As expected, we found a striking divergence in expression profiles of cells exposed to OSS vs. those maintained under static conditions. More genes were upregulated (compared to *t* = 0h) upon continued culture under static conditions compared with transfer to OSS. Increased expression of a smaller set of genes was selectively observed upon exposure to OSS, and the time course of activation was also variable for different genes. Overall, these data are consistent with the idea that mechanosensitive stimuli trigger rapid changes in gene transcription that contribute to the enhanced differentiation of our PT cells.

### Membrane Trafficking Protein Transcripts Upregulated in OK Cells Exposed to OSS

**FIGURE 6:**
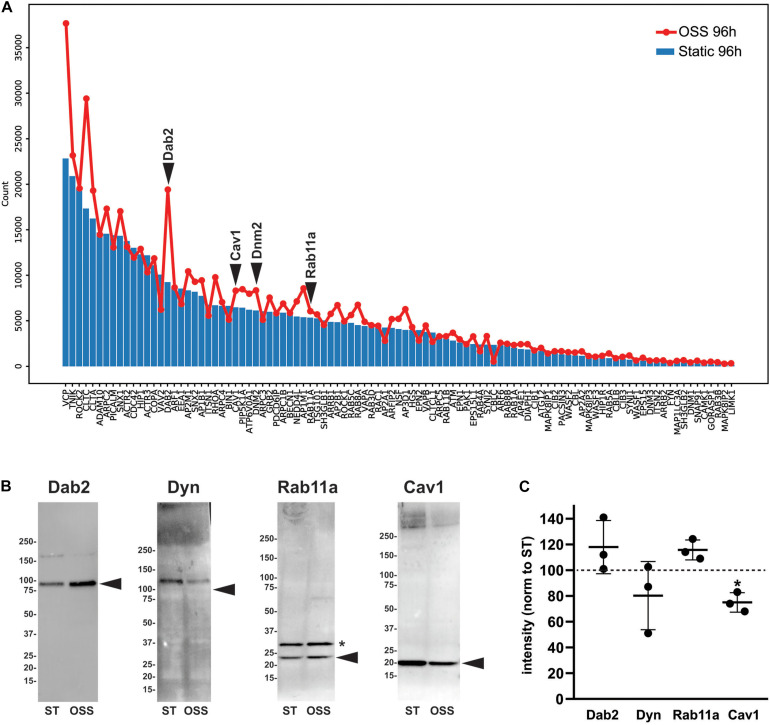
Transcript and protein levels of endocytic traffic proteins in cells cultured under static or OSS conditions. **(A)** RNA transcript expression (average raw reads) of the genes involved in endocytic traffic in cells cultured for 96 h under static (blue bars) or OSS (red line) conditions is plotted in descending order of expression. **(B)** Equivalent amounts of lysates from cells cultured for 96h under static (ST) or OSS conditions were blotted with antibodies against Dab2, Dynamin-2 (Dyn), Rab11a, and Caveolin1 (Cav1). The migration of molecular mass markers is shown on the left, and the arrowheads denote the predicted molecular mass of each protein. The asterisk denotes a band also observed in representative blots by the supplier that is presumed to represent a post-translational modification of Rab11a. **(C)** Band intensities in western blots were quantified and values for 96h OSS (black circles) were normalized to 96 h Static for each protein above (dashed line). Individual values and the mean ± SD of three independent experiments are plotted. *P = 0.029 vs. 96 h Static by one sample *t*-test.

To identify proteins that make up and regulate the elaborate apical endocytic pathway of fully differentiated PT cells, we compared RNA transcript expression levels of a curated list based on the Dharmacon-Thermo Fisher Scientific membrane trafficking siRNA library (Wen et al., 2014). Of the 140 genes on that list, 98 were expressed with an average count of >100 in OK cells. Figure 6A shows the relative expression in cells cultured for 96 h under OSS (red line) vs. static conditions (blue bars). Strikingly, nearly all of the genes were expressed at higher levels in cells cultured under OSS. Among the most dramatic differences in expression was an increase in Dab2, the adaptor protein that mediates megalin and cubilin endocytosis. To assess whether transcriptomic changes correlate with alterations in protein levels we western blotted equivalent amounts of cell lysates with antibodies against Dab2, Dynamin-2 (encoded by *DNM2*), and Rab11a (Figures 6B,C). Whereas Dab2 and Rab11a protein levels appeared slightly increased, reduced levels of dynamin-2 tended to be reduced in cells cultured under OSS. We also blotted lysates to detect caveolin-1 (encoded by *CAV1*), a component of caveolae known to be expressed in PT cells in culture but absent from PT *in vivo* (Zhuang et al., 2011). Whereas *CAV1* transcripts were increased in cells cultured under OSS, caveolin-1 protein levels were considerably lower in these cells compared with cells maintained under static conditions (Figures 6B,C).

## Discussion

Dissecting the PT-specific injury and repair mechanisms using *in vivo* models is challenging because of the multiple cell types that inhabit the kidney and difficulties accessing post-glomerular kidney segments. The availability of a well-characterized, optimally differentiated cell culture model that recapitulates PT function and gene expression is thus of paramount importance to complement whole animal studies Our comparative transcriptional analysis here was directed toward addressing the following questions: What pathways are turned on/off upon progressive culture under OSS or static conditions? Are cells cultured under OSS transcriptionally more representative of the PT *in vivo*? Which transcriptional pathways are triggered by mechanosensitive cues vs. improved oxygenation in our culture model? And, can we identify transcriptional changes in membrane trafficking proteins that underlie the enhanced apical endocytic capacity of cells cultured under OSS?

Using standard cutoff criteria, we found no genes whose expression was significantly upregulated upon shifting cells to OSS for 12 h, whereas expression of 41 genes was downregulated. These genes were components of pathways related to cell survival, differentiation, transport, protein trafficking, and metabolism. Upon longer incubation at OSS, pathway analysis revealed selective engagement of pathways consistent with reduced migration, enhanced cell adhesion, and establishment of apical and basolateral polarized domains necessary for proper differentiation of the PT. By contrast, pathways associated with kidney disease were selectively de-enriched upon prolonged exposure to OSS, suggesting that cells cultured under OSS increasingly resemble normally functioning PT cells. Consistent with this, tSNE comparison of our data with the gene expression profile of deep-sequenced rat nephron segments confirmed that OK cells cultured under OSS more closely resembled *in vivo* PTs compared with cells maintained under static conditions.

Our OSS culture condition increases both oxygen availability and mechanical stimulation of our PT cells. We and others have previously demonstrated dramatic changes in metabolism and metabolic enzyme transcription when cells are cultured on rotating or rocking platforms (Cole et al., 1986; Sahai et al., 1989; Gstraunthaler et al., 1999; Ren et al., 2019). For example, consistent with loss of hypoxic stimuli under OSS, our initial list of significantly downregulated transcripts in cells cultured under OSS for 12 h includes adrenomedullin, a peptide that suppresses EMT in hypoxic PT cells (Zhu et al., 2015). However, we also found that spatial changes in endocytic capacity and Na^+^/K^+^-ATPase expression conform to the profile of shear stress experienced by cells at increasing distances from the center of rotation (Ren et al., 2019). Similarly, it is likely that transcriptional changes vary along the radius of the transwell, although we were unable to test this directly. It is also possible that exposure to linear flow as experienced *in vivo* rather than the rotational flow used here might further improve differentiation of this cell culture model.

Because there is a significant intersection between mechanosensitive and metabolically-activated pathways (Niu et al., 2019), we attempted to differentiate the temporal changes in transcription resulting from mechanosensitive cues vs. oxygenation by analyzing our data against a curated set of genes and pathways altered when renal PT cells were exposed to shear stress using a cone-plate device (Kunnen et al., 2018). Unlike our culture model, cells rotated under this device are not expected to experience dramatically improved oxygen availability (Malek et al., 1993). Querying these pathways, we found a striking divergence in transcript profiles of cells exposed to OSS vs. those maintained under static conditions. These changes included components of the Wnt/β-catenin, TGF-β, and MAP/ERK signaling pathways, which have previously been shown to regulate mechanosensitive signaling in numerous cells and organ systems, including the lymphatic vasculature, kidney, and chondrocytes. (Cha et al., 2016; Szeto et al., 2016; Subramanian et al., 2017). We also observed rapid and sustained decreases in transcripts encoding integrin α9 (ITGA9) and lysyl oxidase like 2 (LOXL2), proteins involved in modulating extracellular matrix components. Such changes may contribute to the differences in migratory/adhesive behavior between cells cultured under OSS vs. static conditions suggested by our pathway analysis. In addition to corroborating the involvement of signaling pathways previously identified as mechanosensitive responses to shear stress, our data also provides temporal information about the sequence of activation. For example, while TP53, CTNNBIP1, and ESR1 in the WNT pathway all show marked expression increases under shear stress, maximal TP53 expression is observed at 12 h, CTNNBIP at 48 h, and ESR1 at 96 h.

Identifying the protein machinery that modulates membrane density of apical receptors and ion transporters is critical for understanding how PT function is regulated, yet the components and compartments that comprise the PT endocytic trafficking pathway remain poorly annotated. Our comparative study identified several proteins associated with the endocytic pathways whose expression is transcriptionally altered upon exposure of cells to OSS for 96 h. Among the most striking difference in expression was an increase in Dab2, the adaptor protein that mediates megalin and cubilin endocytosis. Transcripts for other proteins implicated in receptor-mediated endocytosis and degradation were also apparently increased, including clathrin heavy chain (CLTC), adaptor protein complex 2 beta subunit (AP2B), CALM (PICALM), a phosphatidylinositol binding protein involved in clathrin assembly, phosphatidylinositol 5-kinase (PIP5K1A), Rab11a, and Rab8a, the ubiquitin ligase NEDD4 (NEDD4L), and the vacuolar ATPase V0 subunit A1 (ATP6V0A1). Western blotting for Dab2 and Rab11a confirmed an increase in protein levels, consistent with the dramatic increase in endocytic membrane flux in cells cultured under OSS (Long et al., 2017). However, despite increased message levels, we found reduced levels of caveolin-1 (CAV1) in cells cultured under OSS.

In summary, our analysis of this dataset confirms that the transcriptional profile of OK cells cultured under continuous OSS more closely resembles that of PTs *in vivo* compared with the standard approach of maintaining cells under static conditions. While mRNA abundance generally correlates well with protein levels at steady state (Lee et al., 2015; Bludau and Aebersold, 2020; Limbutara et al., 2020), our data almost certainly underestimate the changes in cellular protein expression that result from cell culture under OSS, as myriad post-transcriptional regulatory pathways also impact protein abundance. Whether the differentiation pathways engaged by OK cells cultured under OSS cells mimic that of embryonic PT cells *in vivo* as the nephron segment matures remains to be resolved. It is also interesting to speculate that our cells may recapitulate differentiation pathways used by PTs undergoing repair after AKI, when tubular flow is reinitiated and O_2_ availability is improved. In particular, OK cells cultured under OSS provide some advantages over organoid cultures as a promising model system for pharmacologic and genomic screens to identify targets that enhance PT function.

## Data Availability Statement

The datasets presented in this study can be found in online repositories. The names of the repository/repositories and accession number(s) can be found below: https://www.ncbi.nlm.nih.gov/geo/, GSE155315.

## Author Contributions

QR, MG, YR, and KL performed experiments. ZF, YB, QR, MG, NR, AP, JL, HP, and OW analyzed data. HP and OW designed the studies and wrote the manuscript. All authors contributed to the article and approved the submitted version.

## Conflict of Interest

The authors declare that the research was conducted in the absence of any commercial or financial relationships that could be construed as a potential conflict of interest.

## Supplementary Material

The Supplementary Material for this article can be found at: https://www.frontiersin.org/articles/10.3389/fphys.2020.587358/full#supplementary-material and on FigShare at: https://figshare.com/s/34052adaa7af8ec8b67b

Supplementary Figure 1 Temporal gene expression changes in cytoskeleton-, matrix-, and cholesterol-related genes. Heat maps of the gene expression profile of gene sets defined using keywords **(A)** Cytoskeleton, **(B)** Cellular Matrix and **(C)** Cholesterol are plotted for cells incubated for 96, 48, and 0 h under Static conditions or 12, 48, and 96 h under OSS.

Supplementary Table 1 RNAseq analysis. Each experimental condition was analyzed from 3 replicate libraries. *p* values were calculated using a *T*-test comparison of Static and OSS values at 96 h. q values were calculated by the method of Benjiamini and Hochberg. Color coded ratios show gene expression comparing each condition to Static 0 h or Static 96 h.

Supplementary Table 2 IPA analysis of significantly differentially-expressed genes in 12 h OSS vs. 0 h Static against IPA *Disease and Biological Function* terms.

Supplementary Table 3 IPA Comparison Analysis of significantly differentially-expressed genes during the course of the exposure to OSS (96 h vs. 48 h and 48 h vs. 12 h) against IPA *Disease and Biological Function* terms.

Supplementary Table 4 Significantly differentially-expressed genes in 96 h OSS vs. 96 h Static calculated using edgeR.

Supplementary Table 5 IPA Comparison Analysis on significantly differentially-expressed genes between 96 h exposure to OSS vs. 0 h Static and 96 h Static vs. 0 h Static against IPA *Disease and Biological Function* terms.
