# Supplementary figures and images for: Transcriptional Programs Driving Shear Stress-Induced Differentiation of Kidney Proximal Tubule Cells in Culture
Source: Front Physiol. 2020 Oct 30;11:587358. doi: 10.3389/fphys.2020.587358 (PMC7662153; doi:10.3389/fphys.2020.587358)

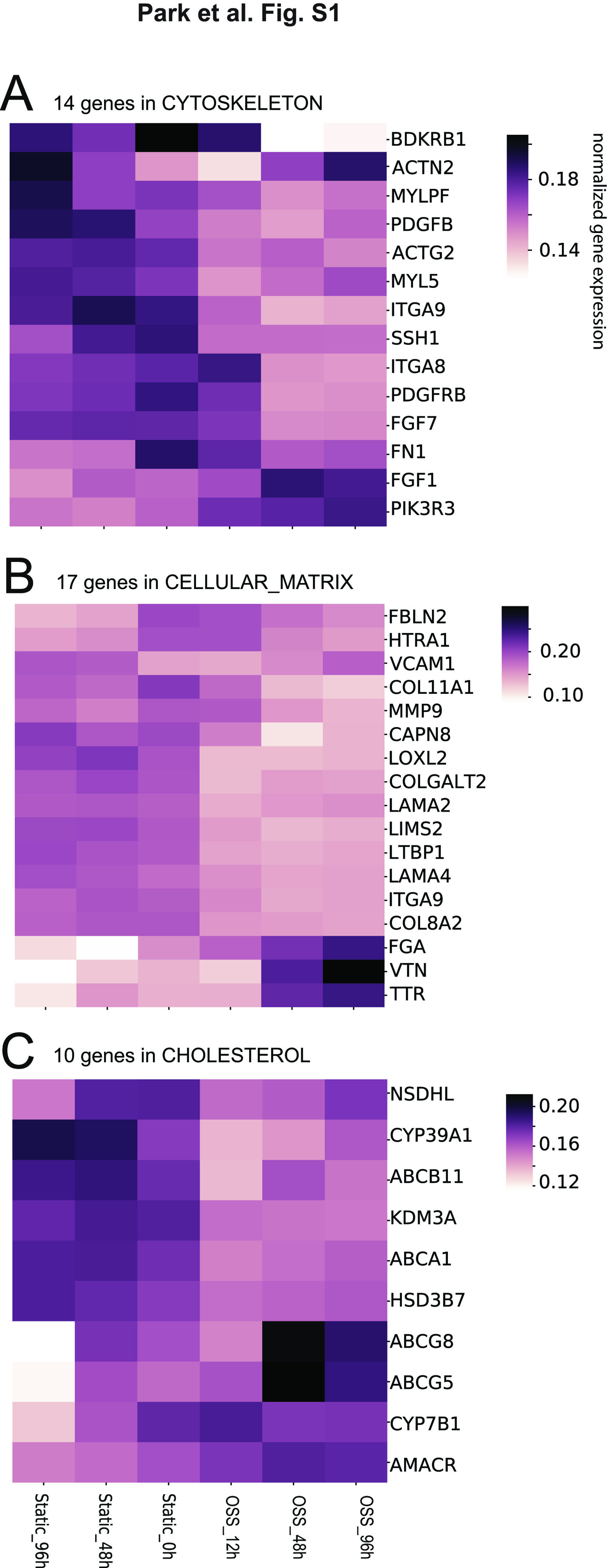

Supplement: Supplementary Figure 1 — Temporal gene expression changes in cytoskeleton-, matrix-, and cholesterol-related genes. Heat maps of the gene expression profile of gene sets defined using keywords (A) Cytoskeleton, (B) Cellular Matrix and (C) Cholesterol are plotted for cells incubated for 96, 48, and 0 h under Static conditions or 12, 48, and 96 h under OSS. [file Image_1.JPEG]
